# Risk of labour market marginalisation among young refugees and non-refugee migrants with common mental disorders

**DOI:** 10.1007/s00127-020-02022-4

**Published:** 2021-01-20

**Authors:** D. Di Thiene, Magnus Helgesson, S. Rahman, K. Alexanderson, J. Tiihonen, G. La Torre, E. Mittendorfer-Rutz

**Affiliations:** 1 Division of Insurance Medicine, Department of Clinical Neuroscience, Karolinska Institutet, SE-171 77 Stockholm, Sweden; 2 Department of Clinical Neuroscience, Centre for Psychiatric Research, Karolinska Institutet, SE-171 77 Stockholm, Sweden; 3 Stockholm Health Care Services, Stockholm County Council, SE-171 77 Stockholm, Sweden; 4 Department of Forensic Psychiatry, Niuvanniemi Hospital, University of Eastern Finland, 70240 Kuopio, Finland; 5 Department of Public Health and Infectious Diseases, Sapienza University of Rome, 00185 Rome, Italy

**Keywords:** Migration, Common mental disorders, Unemployment, Sickness absence, Duration of residence, Age at arrival

## Abstract

**Purpose:**

Labour market marginalisation (LMM), i.e. long-term unemployment (LTU), long-term sickness absence (LTSA) and disability pension (DP), among young individuals with common mental disorders (CMDs) are a challenge for the welfare system, and refugees and non-refugee migrants seem particularly vulnerable. The aim was to investigate the risk of LMM in young adults with CMDs among refugees and non-refugee migrants compared to Swedish-born individuals and the role of country of birth, duration of residence and age at arrival.

**Methods:**

A prospective cohort study was conducted including young adults (19–30 years) with inpatient or specialised outpatient healthcare due to CMDs and/or antidepressant prescriptions during 2009 (*N* = 69,515). Cox regression models were used to calculate hazard ratios (HRs) with 95% confidence intervals for the risk of LMM during 2010–2013.

**Results:**

Both refugees and non-refugee migrants had a higher risk of LTU compared to Swedish-born individuals (HR refugees: Africa: 2.4; Asia: 2.2; Europe outside EU25: 1.6; South America: 1.4) with highest estimates in refugees from Afghanistan and Syria. Refugees from Africa and Asia had a lower risk of LTSA compared to Swedish-born individuals (HR: 0.6 and 0.7, respectively), particularly refugees from Afghanistan and Iraq. Especially among refugees, a longer duration of residence and a younger age at arrival were associated with a lower risk of LTU.

**Conclusions:**

The risk of LTU among refugees and non-refugee migrants was higher and the risk of LTSA was lower, compared to Swedish-born individuals. Duration of residence and age at arrival had an influence on the risk of LTU, particularly among refugees.

**Supplementary Information:**

The online version contains supplementary material available at 10.1007/s00127-020-02022-4.

## Background

Nowadays, labour market marginalisation constitutes a considerable public health problem in a number of countries of the Organisation for Economic Co-operation and Development (OECD), especially among young adults [1–3]. This implies an economic challenge for welfare states and adverse social and mental consequences for young individuals [1, 2]. Labour market marginalisation can be defined in different ways, a comprehensive definition of labour market marginalisation includes both unemployment, and measures based on medical assessments, such as sickness absence and disability pension, to have a better understanding of the phenomenon [4–6]. In fact, previous research suggested that social consequences of mental ill-health can be underestimated when labour market marginalisation is only defined as unemployment [7, 8].

Regarding labour market marginalisation, migrants are one of the most vulnerable populations. During the last decades, Sweden has experienced an increase of migrants and today around a fifth of the population in Sweden is born abroad [9]. Migrants are reported to have more problems in finding and keeping a job compared to Swedish-born individuals [6, 10, 11]. Furthermore, time-related factors, such as duration of residence in the new country and the age at arrival, were shown to be associated with the risk of labour market marginalisation [11, 12]. Some studies reported a lower level of unemployment with increasing number of years of residence in the host country, others a better integration with a younger age at arrival [11, 13].

Common mental disorders, defined as either depressive, anxiety or stress-related disorders, are among the main causes for labour market marginalisation in countries within the OECD [3]. This is particularly seen among young adults and is in part attributable to the early age of onset and the high prevalence of relapses among these disorders [14, 15]. Furthermore, the existence of a reverse causation process has been shown, where common mental disorders in turn increase the risk of labour market marginalisation and, on the other side, being marginalised at the labour market can lead to common mental disorders [7, 16, 17]. This can be particularly harmful in young individuals, when difficulties in entering the labour market can play an additional role in the risk of marginalisation [18]. Migrants were reported to have a higher risk of common mental disorders and refugees were particularly vulnerable [7, 19]. Factors related to pre-migration, such as traumatic events, and postmigration-related aspects such as discrimination in the host country, can have consequences on the employment outcomes [20–22]. Since the number of refugees has increased sharply during the last decades, research regarding mental disorders and social integration among refugees is warranted [23, 24]. This is internationally the first study to investigate specific risk groups of refugees with common mental disorders with regard to subsequent labour market marginalisation.

## Aims

The aim of this study was to investigate: (1) the risk of labour market marginalisation, measured as long-term unemployment, long-term sickness absence and disability pension, among young refugees, non-refugee migrants and Swedish-born individuals with common mental disorders and (2) the role of country of birth, duration of residence and age at arrival for the risk of labour market marginalisation among refugees and non-refugee migrants.

## Methods

### Study population

This population-based prospective cohort study consisted of all individuals aged 19–30 years, resident in Sweden in 2009, who during 2009 had: (a) inpatient or specialised outpatient healthcare due to common mental disorders or (b) prescribed antidepressants (*n* = 83,769). Common mental disorders were measured as a main diagnosis of either a depressive, anxiety, or stress-related disorder according to the International Classification of Diseases version 10 (ICD-10) codes F32, F33, or F40-F43. Prescribed antidepressants were defined by the code N06A according to the Anatomical Therapeutic Chemical Classification System **(**ATC). Individuals on disability pension in 2009 (*n* = 11,616) and with missing data on reason for settlement (e.g. refugee status) in Sweden (*n* = 2638) were excluded. The final study population included 69,515 individuals where 26,593 individuals were included due to a diagnosis from inpatient or specialised outpatient healthcare and 42,922 individuals were included from having been prescribed antidepressants. There was a 26% overlap between individuals identified by specialised healthcare records and those identified by antidepressant prescription.

### Exposure variables

Refugees were defined as: (1) persons who were granted asylum in Sweden due to reasons stipulated by the Geneva Convention [25], (2) persons in need of protection who were granted asylum in Sweden due to other reasons, and also resettled refugees arriving to Sweden on request by United Nations High Commissioner for Refugees (UNHCR) and (3) persons who were granted asylum due to humanitarian reasons [6, 25]. Seven countries, that generated the highest number of refugees to Sweden up until 2009, were specifically analysed: Somalia, Afghanistan, Iraq, Iran, Syria, former Yugoslavia and Chile. Additionally, we considered the following regions of birth covering all included countries: Africa, Asia, Europe outside EU25 and South America. Non-refugee migrants were defined as individuals born outside Sweden with a reason for settlement e.g. labour, study, or family reunification. The division of refugees and non-refugee migrants can give valuable information if traumatic events or the influence of culture and religion is the driving force for labour market marginalisation. Swedish-born individuals were defined as individuals born in Sweden and build the reference groups for analyses in Table 2. Moreover, two additional variables related to immigration were used as exposure variables: (1) duration of residence in Sweden: <10 and ≥10 years; (2) age at arrival in Sweden ≤ 16 or > 16 years.

### Register data

Nationwide register data, linked at individual level (based on the unique personal number assigned to all residents in Sweden), from the following authorities were used: (1) Statistics Sweden: Longitudinal Integration Database for Health Insurance and Labour Market Studies (LISA) for age, sex, type of living area, educational level, family situation, country of birth, number of annual days of receiving unemployment benefits, year of immigration and emigration; Longitudinal database for integration studies (STATIV) for information on reason for settlement in Sweden; (2) Social Insurance Agency: information on date, number of days and grade of sickness absence and disability pension; (3) the National Board of Health and Welfare: National patient register for date and cause of inpatient and specialised outpatient healthcare; cause of death register for information on date of death; prescribed drug register for information on prescribed psychiatric medication.

### Sickness absence, disability pension and unemployment

In Sweden, individuals aged 20–64 years who are enrolled at the Public Employment Service, are ready to enter the labour market at any time and have a job-seeking plan, are covered by the basic insurance and can receive unemployment benefits, even without previous work experience. Additionally, it is possible to have a voluntary income-loss insurance with a requirement to have a minimum of 6 months of employment to be entitled to such unemployment benefits. Regarding sickness absence, individuals from the age of 16 with income from work or unemployment benefits are covered by the public sickness absence scheme and can claim sickness absence benefits if they have reduced work capacity due to disease or injury at least for 25% [26]. All individuals, 30–64 years, can be granted permanent disability pension if the work capacity is permanently reduced due to morbidity. Individuals aged 19–29 years can be granted temporary disability pension if their work capacity is reduced at least for 25%, or if compulsory or upper secondary school is not finished in due time [27]. Both sickness absence and disability pension can be granted for full- or part-time at 25, 50 or 75%. Sickness absence benefits cover 80% and disability pension 65% of lost income up to a certain level. Immigrants are entitled to enter the labour market and have access to social insurance benefits given the abovementioned eligibility criteria after receiving a residence permit.

### Outcome measures

Three different outcome measures during 2010–2013 were used: (1) long-term unemployment (> 180 days annually), (2) long-term sickness absence (> 90 net days annually), and (3) disability pension.

### Covariates

Covariates in the analyses were (1) sociodemographic variables: sex, age (19–24 years; 25–30 years); educational level (elementary (0–9 years), high school (10–12 years), university/college (> 12 years) and missing); family situation (married/cohabitant without children living at home, married/cohabitant living with children, single/divorced/separated/widowed without children living at home, single/divorced/separated/widowed with children living at home, young adults (19–20 years old) living with parents); type of living area (big cities, medium-sized cities and small cities/villages); (2) variables related to work: labour market attachment (income from work, income not from work and no income); (3) variables related to health: comorbid somatic disorders, measured as in- or specialised outpatient healthcare 2009 due to somatic disorders (including all diagnoses except (ICD-10 codes F00-99). All these variables were measured at baseline.

### Statistics

After testing if the proportional hazards assumption was met by visually comparing Kaplan–Maier curves, Cox proportional hazard regression models were used to calculate crude and adjusted hazard ratios (HRs) with 95% confidence intervals (CIs) for the risk of subsequent long-term unemployment, long-term sickness absence and disability pension during 2010–2013. HRs were calculated with different reference groups: in Table 2, Swedish-born individuals; in Table 3, the lower category of duration of residence from the same region of birth; in Table 4 the higher category of age at arrival from the same region of birth. Censoring was done in case of emigration, death, end of follow-up, whichever occurred first. For the analyses, in which long-term unemployment and long-term sickness absence were outcome measures, censoring was also due to disability pension. All multivariate analyses were adjusted for age, sex, educational level, family situation, type of living area, labour market attachment and comorbid somatic disorders. SPSS version 20.0 was used for all analyses.

## Results

In all the three groups (i.e. refugees, non-refugee migrants and Swedish-born individuals), there was a higher proportion of women, particularly among Swedish-born individuals (64.6%) (Table 1). Swedish-born individuals had a higher proportion of young individuals (45.9% in the 19–24 years’ group) compared to refugees (38.4%) and non-refugee migrants (34.5%). Refugees had more often a low educational level (32.7%) compared to non-refugee migrants (28.0%) and Swedish-born individuals (21%). A higher proportion of non-refugee migrants (59.9%) had a duration of residence > 10 years compared to refugees (36.3%), whereas refugees were more often young (0–16 years) when arriving in Sweden compared to non-refugee migrants (65.9 vs 42.2%, respectively).

**Table 1:** Sociodemographic characteristics of individuals, 19–30 years old, resident in Sweden with a common mental disorder (according to a diagnosis in specialised healthcare and/or prescription of antidepressants) during 2009

All	Swedish-born individuals *n* (%)	Refugees^b^ *n* (%)	Non-refugee migrants^c^ *n* (%)
Sex			
Men	22,249 (35.4)	1335 (46.1)	1339 (36.3)
Women	40,687 (64.6)	1560 (53.9)	2345 (63.7)
Age (years)			
19–24	28,882 (45.9)	1112 (38.4)	1272 (34.5)
25–30	34,054 (54.1)	1783 (61.6)	2412 (65.5)
Educational level (years)			
Low (0–9)	13,296 (21.1)	947 (32.7)	1030 (28.0)
Medium (10–12)	31,103 (49.4)	1114 (38.5)	1375 (37.3)
High (> 12)	18,318 (29.1)	659 (22.8)	1048 (28.4)
Missing	219 (0.3)	175 (6.0)	231 (6.3)
Family situation			
Married/living with partner without children^d^	1340 (2.1)	185 (6.4)	362 (9.8)
Married/living with partner with children^d^	7443 (11.8)	512 (17.7)	879 (23.9)
Single/divorced/separated/widowed without children^d^	44,919 (71.4)	1841 (63.6)	1961 (53.2)
Single/divorced/separated/widowed with children^d^	3243 (5.2)	202 (7.0)	316 (8.6)
Young adults (≤ 20 years old) living at home	5991 (9.5)	155 (5.4)	166 (4.5)
Type of living area^a^			
Big cities	24,566 (39.0)	1316 (45.5)	2053 (55.7)
Medium-sized cities	23,497 (37.3)	1137 (39.3)	1159 (31.5)
Small cities/villages	14,873 (23.6)	442 (15.3)	471 (12.8)
Duration of residence			
0–5 years	n.a	801 (27.6)	1537 (41.8)
6–9 years	n.a	359 (12.4)	806 (21.9)
≥ 10 years	n.a	1735 (59.9)	1338 (36.3)
Age at arrival			
0–16 years	n.a	1886 (65.9)	1548 (42.2)
17–30 years	n.a	978 (34.1)	2119 (57.8)
Specific countries (% in row)			
Asia without Afghanistan, Iran, Iraq and Syria	n.a	307 (28.6)	764 (71.4)
Afghanistan	n.a	115 (54.5)	96 (45.5)
Iran	n.a	378 (54.9)	311 (45.1)
Iraq	n.a	563 (54.8)	464 (45.2)
Syria	n.a	76 (50.0)	76 (50.0)
Africa without Somalia	n.a	106 (32.9)	216 (67.1)
Somalia	n.a	98 (53.3)	86 (46.7)
South America without Chile	n.a	43 (18.8)	186 (81.2)
Chile	n.a	88 (47.8)	96 (52.2)
Europe outside EU25 (without Former Yugoslavia)	n-a	121 (28.4)	610 (71.6)
Former Yugoslavia	n.a	952 (74.0)	335 (26.0)
Other countries	n.a	48 (9.7)	444 (90.2)
Labour market attachment			
Income from work	33,408 (53.1)	994 (34.4)	1344 (36.5)
Income, not from work	15,520 (24.6)	656 (22.6)	822 (22.3)
No income	14,008 (22.3)	1245 (43.0)	1518 (41.2)
Specialised healthcare due to somatic disorders (2009)			
Yes	26,854 (42.7)	1383 (47.7)	1795 (48.7)
No	36,082 (57.3)	1512 (52.3)	1889 (51.3)

^a^Type of living area: big cities: Stockholm, Gothenburg and Malmö; medium-sized cities: cities with more than 90,000 inhabitants within 30 km distance from the centre of the city; small cities/villages

^b^Refugee status: immigrants with reason of settlement as refugee, humanitarian reasons, need of protection

^c^Migrant with reason for settlement as labour, study or family reunification

^d^Children living at home

### Long-term unemployment

Both refugees and non-refugee migrants had a higher risk of long-term unemployment compared to Swedish-born individuals (Table 2). According to region of birth, the adjusted risk estimates of long-term unemployment were two times higher for refugees from Africa, Asia and Europe outside EU25 (HR: 2.39, HR: 2.16 and HR: 1.61, respectively). According to the country of birth, the highest risk estimates for long-term unemployment were observed for refugees from Afghanistan (HR: 2.65), Somalia (HR: 2.49), Syria (HR: 2.58) and Iraq (HR: 2.36). HRs among non-refugee migrants were similar to refugees, but on a slightly lower level.

**Table 2:** Crude and multivariate hazard ratios (HRs) with 95% confidence intervals (CIs) for long-term unemployment, long-term sickness absence (SA) and disability pension (DP) during 2010–2013 among 19–30 years old refugees and non-refugee immigrants (according to region and country of birth), with a common mental disorder (diagnosis and/or prescription of antidepressant) during 2009 compared with Swedish-born individuals of similar age

	Long-term unemployment		Long-term sickness absence		Disability pension	
	Crude HR (95% CI)	Adjusted^a^ HR (95% CI)	Crude HR (95% CI)	Adjusted^a^ HR (95% CI)	Crude HR (95% CI)	Adjusted^a^ HR (95% CI)
Swedish-born individuals^b^	1	1	1	1	1	1
Refugees^c^						
Africa	3.72 (2.97–4.66)	2.39 (1.91–3.05)	0.55 (0.33–0.89)	0.60 (0.36–0.98)	0.90 (0.48–1.68)	0.60 (0.32–1.11)
Somalia	4.09 (3.03–5.59)	2.49 (1.80–3.40)	0.58 (0.29–1.16)	0.60 (0.30–1.20)	1.13 (0.51–2.53)	0.65 (0.28–1.41)
Asia	3.12 (2.85–3.42)	2.16 (1.96–2.36)	0.68 (0.58–0.81)	0.72 (0.61–0.85)	0.70 (0.54–0.91)	0.50 (0.38–0.65)
Afghanistan	3.91 (2.93–5.21)	2.65 (1.98–3.53)	0.29 (0.12–0.71)	0.35 (0.14–0.84)	0.63 (0.23–1.33)	0.37 (0.14–1.01)
Iraq	4.12 (3.62–4.69)	2.36 (2.06–2.68)	0.37 (0.26–0.54)	0.39 (0.26–0.53)	0.81 (0.54–1.20)	0.47 (0.31–0.69)
Iran	2.06 (1.66–2.54)	1.82 (1.46–2.24)	0.97 (0.74–1.27)	1.08 (0.76–1.34)	0.43 (0.22–0.83)	0.45 (0.23–0.87)
Syria	4.01 (2.83–5.66)	2.58 (2.20–4.42)	1.16 (0.63–1.96)	0.97 (0.50–1.75)	0.71 (0.23–2.22)	0.54 (0.17–1.68)
Europe outside EU25	1.99 (1.75–2.27)	1.61 (1.42–2.01)	1.02 (0.86–1.19)	0.99 (0.86–1.19)	0.70 (0.54–0.91)	0.78 (0.59–1.01)
F. Jugoslavia	1.93 (1.68–2.21)	1.56 (1.35–1.78)	1.02 (0.86–1.21)	0.98 (0.81–1.14)	0.98 (0.74–1.29)	0.79 (0.60–1.04)
South America	1.67 (1.13–2.48)	1.36 (0.91–2.01)	1.16 (0.77–1.78)	1.15 (0.77–1.83)	0.27 (0.69–1.10)	0.26 (0.07–1.06)
Chile	1.58 (0.96–2.58)	1.20 (0.73–1.96)	1.41 (0.90–2.21)	1.35 (0.86–2.12)	0.56 (0.18–1.74)	0.49 (0.12–1.93)
Non-refugee migrants^d^						
Africa	3.04 (2.49–3.71)	2.13 (1.74–2.59)	0.72 (0.50–1.02)	0.72 (0.50–1.02)	0.36 (0.16–0.80)	0.24 (0.10–0.54)
Somalia	3.76 (2.67–5.30)	2.38 (1.68–3.35)	0.49 (0.22–1.09)	0.52 (0.22–1.16)	0.42 (0.10–1.71)	0.20 (0.52–0.83)
Asia	2.71 (2.47–2.96)	2.01 (1.82–2.18)	0.70 (0.60–0.81)	0.68 (0.60–0.81)	0.73 (0.57–0.93)	0.54 (0.42–0.68)
Afghanistan	2.09 (1.39–3.15)	1.36 (0.90–2.05)	0.49 (0.23–0.64)	0.48 (0.22–1.01)	0.18 (0.26–0.89)	0.12 (0.18–0.89)
Iraq	3.48 (2.99–4.05)	2.29 (1.96–2.67)	0.63 (0.47–0.86)	0.58 (0.41–0.75)	0.79 (0.51–1.22)	0.44 (0.28–0.68)
Iran	2.70 (2.19–3.30)	2.20 (1.77–2.70)	0.71 (0.50–1.01)	0.72 (0.50–1.02)	0.77 (0.45–1.33)	0.70 (0.39–1.21)
Syria	3.52 (2.44–5.07)	2.19 (1.50–3.13)	0.92 (0.49–1.71)	0.80 (0.40–1.40)	0.95 (0.35–2.53)	0.67 (0.24–1.81)
Europe outside EU25	2.49 (2.11–2.94)	2.02 (1.82–2.19)	1.01 (0.80–1.27)	0.95 (0.75–1.19)	0.79 (0.52–1.20)	0.70 (0.46–1.05)
F. Jugoslavia	2.72 (2.23–3.33)	1.94 (1.58–2.36)	1.13 (0.86–1.48)	0.95 (0.71–1.22)	0.93 (0.58–1.50)	0.83 (0.51–1.34)
South America	2.09 (1.64–2.66)	1.99 (1.56–2.54)	0.73 (0.50–1.05)	0.76 (0.53–1.10)	1.10 (0.68–1.77)	0.98 (0.61–1.59)
Chile	2.85 (1.99–4.08)	2.43 (1.73–3.55)	0.64 (0.33–1.24)	0.61 (0.31–1.20)	0.41 (0.10–1.64)	0.53 (0.17–1.68)

^a^Adjusted for age, sex, educational level, family situation, type of living area, labour market attachment in 2009 and inpatient and specialised outpatient healthcare due to somatic disorders during 2009

^b^Native Swedes is the reference group

^c^Refugee status: immigrants with reason of settlement as, refugee, humanitarian reasons, need of protection

^d^Immigrants with reason of settlement due to labour, study, family reunification

### Long-term sickness absence and disability pension

Refugees from Africa or Asia had lower adjusted HRs for long-term sickness absence compared to Swedish-born individuals (HR: 0.60 and 0.72, respectively). According to the country of birth, the estimates for long-term SA were significantly lower for refugees from Afghanistan (HR: 0.35) and Iraq (HR: 0.39), and for non-refugee migrants from Iraq (HR: 0.58). The risk of disability pension was lower for refugees from Asia (HR: 0.50) and for non-refugee migrants from Africa (HR: 0.24) and Asia (HR: 0.54) compared to Swedish-born individuals.

### Duration of residence and age at arrival

Among refugees from Africa and Asia, a duration of residence in Sweden ≥ 10 years was associated with a lower risk of long-term unemployment, compared to the refugees of the same continent with a duration of residence less than 10 years (Table 3). In the same groups, the risk of long-term sickness absence was higher for non-refugee migrants from Africa and Asia compared to non-refugee migrants from the same region of birth with a duration of residence < 10 years. Refugees and non-refugee migrants from Asia with a duration of residence of ≥ 10 years showed also a higher risk of disability pension (HR: 1.87 and 1.83, respectively) compared to their counterparts with < 10 years. Refugees from Africa (HR:0.50) and Asia (HR:0.66) that arrived in Sweden at age ≤ 16 years had a lower risk of long-term unemployment compared to refugees from the same regions of birth with an age at arrival >16 years (Table 4). With regard to long-term sickness absence, non-refugee migrants from Asia and Africa arriving ≤ 16 years old showed lower risk estimates compared to those with the same region of birth whose age at arrival in Sweden was > 16 years.

**Table 3:** Multivariate hazard ratios (HRs) with 95% confidence intervals (CIs) for long-term unemployment, long-term sickness absence (SA) and disability pension (DP) during 2010–2013 among refugees and non-refugee migrants resident in Sweden (19–30 years old), with a common mental disorder (diagnosis and/or prescription of antidepressant) during 2009 and with a duration of residence in the country ≥ 10 years compared with refugees and non-refugee migrants with a duration of residence < 10 years from the same region of birth

	Long-term unemployment		Long-term sickness absence		Disability pension	
	Crude HR (95% CI)	Adjusted^a^ HR (95% CI)	Crude HR (95% CI)	Adjusted^a^ HR (95% CI)	Crude HR (95% CI)	Adjusted^a^ HR (95% CI)
Duration of residence < 10 years^b^	1	1	1	1	1	1
Refugees^c^ with residence ≥ 10 years						
Africa	0.54 (0.33–0.88)	0.60 (0.36–1.01)	3.07 (1.06–8.84)	1.78 (0.56–5.26)	0.69 (0.17–2.76)	1.06 (0.24–4.94)
Asia	0.52 (0.43–0.62)	0.68 (0.55–0.84)	2.39 (1.68–3.41)	1.37 (0.91–2.01)	0.95 (0.56–1.62)	1.87 (1.05–3.32)
South America	0.87 (0.32–2.32)	0.92 (0.31–2.68)	1.50 (0.44–5.09)	0.69 (0.19–2.54)	n.a^d^	n.a
Europe outside EU25	0.85 (0.62–1.16)	1.07 (0.77–1.49)	1.15 (0.74–1.76)	0.86 (0.56–1.38)	0.68 (0.36–1.28)	0.96 (0.50–1.83)
Non-refugee migrants^e^ with residence ≥ 10 years						
Africa	0.74 (0.49–1.14)	0.87 (0.55–1.33)	3.32 (1.55–7.11)	3.17 (1.43–7.10)	1.54 (0.31–7.65)	1.74 (0.31–9.71)
Asia	0.64 (0.53–0.76)	0.76 (0.63–0.93)	2.22 (1.62–3.02)	1.83 (1.31–2.54)	1.53 (0.95–2.46)	1.83 (1.09–3.04)
South America	0.71 (0.44–1.17)	0.95 (0.54–1.58)	1.37 (0.64–2.90)	1.41 (0.64–3.10)	3.88 (1.17–13.53)	2.97 (0.78–11.40)
Europe outside EU25	0.75 (0.52–1.08)	0.91 (0.61–1.32)	1.56 (0.98–2.47)	1.78 (1.08–2.96)	1.24 (0.53–2.86)	1.39 (0.58–3.37)

^a^The model was adjusted for age, sex, educational level, family situation, type of living area, labour market attachment in 2009 and specialised healthcare due to somatic disorders during 2009

^b^The reference group comprised refugees and non-refugee migrants with a duration of residence < 10 years from the same region of birth

^c^Refugee status: migrants with a reason of settlement as refugee according to Geneva Convention, humanitarian reasons or in need of protection

^d^n.a. not applicable

^e^Non-refugee migrants: migrants with reason of settlement as labour, study or family reunification

**Table 4:** Crude and multivariate hazard ratios (HRs) with 95% confidence intervals (CIs) for long-term unemployment, long-term sickness absence and disability pension during 2010–2013 among refugees and non-refugee migrants from different regions of birth and resident in Sweden (19–30 years old), with a common mental disorder (diagnosis and/or prescription of antidepressant) during 2009 with the age at arrival in Sweden ≤ 16 years compared to refugees and non-refugee migrants from the same region of birth and with age at arrival > 16 years

	Long-term unemployment		Long-term sickness absence		Disability pension	
	Crude HR (95% CI)	Adjusted^a^ HR (95% CI)	Crude HR (95% CI)	Adjusted HR (95% CI)	Crude HR (95% CI)	Adjusted HR (95% CI)
Age at arrival > 16 years^b^	1	1	1	1	1	1
Refugees^c^ age at arrival ≤ 16 years						
Africa	0.40 (0.24–0.66)	0.50 (0.29–0.85)	3.62 (1.16–11.22)	2.22 (0.67–7.27)	2.30 (0.57–9.19)	2.75 (0.53–14.19)
Asia	0.48 (0.40–0.58)	0.66 (0.54–0.81)	1.87 (1.32–2.65)	1.16 (0.79–1.70)	0.74 (0.44–1.27)	1.45 (0.81–2.59)
South America	0.68 (0.25–1.82)	0.89 (0.30–2.59)	1.19 (0.35–4.05)	0.97 (0.28–3.36)	n.a^d^	n.a
Europe outside EU25	0.65 (0.48–0.90)	0.90 (0.64–1.25)	1.16 (0.73–1.84)	0.96 (0.59–1.56)	0.79 (0.39–1.57)	0.98 (0.47–2.06)
Non-refugee migrants^e^ age at arrival ≤ 16 years						
Africa	0.73 (0.48–1.11)	0.85 (0.54–1.35)	2.40 (1.14–5.04)	2.68 (1.21–5.94)	2.62 (0.48–14.38)	2.55 (0.37–17.34)
Asia	0.51 (0.42–0.62)	0.64 (0.51–0.79)	1.54 (1.14–2.09)	1.55 (1.10–2.19)	1.51 (0.94–2.43)	1.44 (0.81–2.56)
South America	0.56 (0.34–0.92)	0.79 (0.44–1.39)	1.10 (0.52–2.34)	1.27 (0.56–2.90)	5.08 (1.16–22.22)	3.17 (0.61–16.53)
Europe outside EU25	0.61 (0.42–0.89)	0.77 (0.50–1.17)	1.06 (0.65–1.71)	1.29 (0.76–2.21)	1.48 (0.64–3.37)	1.81 (0.70–4.66)

^a^The model was adjusted for age, sex, educational level, family situation, type of living area, labour market attachment in 2009 and specialised healthcare due to somatic disorders during 2009

^b^The reference group comprised refugees and non-refugee migrants with age at arrival > 16 years from the same region of birth

^c^Refugee status: migrants with reason of settlement as refugee according to the Geneva Convention, humanitarian reasons or in need of protection

^d^n.a. not applicable

^e^Non-refugee migrants: migrants with reason of settlement due to labour, study or family reunification

### Sensitivity analyses

The sensitivity analyses showed comparable results for individuals with a common mental disorders diagnosis from in- or specialised outpatient healthcare and those with prescription of antidepressants. Results were similar for all three refugee groups i.e. refugees according to the Geneva Convention, humanitarian reasons and need of protection.

## Discussion

### Main findings

In this study on young adults with common mental disorders, the risk of long-term unemployment among refugees and non-refugee migrants was higher, compared to Swedish-born individuals and generally higher among refugees, especially from Somalia, Afghanistan, Syria and Iraq. Refugees and non-refugee migrants from Africa and Asia, who lived in Sweden for ≥ 10 years, showed a reduced risk for long-term unemployment compared to their respective counterparts who arrived  <10 years ago. Those who immigrated when ≤ 16 years of age had a lower risk of unemployment compared to those who arrived when > 16 years of age. The risk of long-term sickness absence was instead lower among both refugees and non-refugee migrants from Africa and Asia compared to Swedish-born individuals.

### Long-term unemployment

Compared to Swedish-born individuals, we found a higher risk of long-term unemployment among refugees as well as among non-refugee migrants which is in line with earlier research on both young and adult populations [6, 11, 28–31]. In a comparison with the largest recipients of refugees in the European Union, Sweden stands out as the most liberal country with regard to proving support and training to enhance a labour market entrance for refugees. The authors conclude that the Swedish policies for labour market integration during the study period are comprehensive, ambitious and enhance labour market mobility [32]. Despite these efforts, young migrants with common mental disorders experience long-term unemployment to a higher extent compared to Swedish-born individuals with the same disorders. Psychiatric disorders have been seen to play an additional role in the risk for long-term unemployment in other studies [5, 7, 22]. One important reason for why psychiatric disorders create discrepancies in long-term unemployment between migrants and Swedish-born individuals and between refugees and non-refugee migrants is most likely due to differences in psychiatric healthcare utilisation including utilisation of psychiatric medication [33]. Differences in healthcare utilisation between migrants and the host population are likely to be due to cultural discrepancies with regard to the stigma towards psychiatric disorders and barriers in access to healthcare, especially among migrants from some regions and countries [34, 35]. Other reasons for a higher risk of long-term unemployment among subgroups of migrants may be found in factors important for the labour market. It has been stated that refugees, compared to non-refugee migrants, are not selected according to employment‐related criteria, and their skills might not therefore match local needs on the labour market [29]. At least medium educational level (10–12 years) is often required for jobs in Sweden, and migrants have in general a much lower educational level compared to Swedish-born individuals [36, 37]. The higher proportion of unskilled workers reported among refugees may therefore contribute to the risk of labour market marginalisation [11]. Among migrants with a self-reported high educational level, processes of translating education acquirements from the home country to the system of the host country are often time-consuming and sometimes not feasible. As they therefore often end up in jobs below their formal educational level, this might contribute to high levels of both long-term unemployment as well as general labour market marginalisation as over qualification has been reported as a strong risk factor for health deterioration [38]. Despite efforts with free language training to all migrants, language barriers may still be a reason for discrepancies in long-term unemployment between subgroups of migrants as the training is voluntary, and some groups of migrants are underrepresented in those classes [6, 39]

In a previous study from our research group, we found that the risk of long-term unemployment among refugees with a common mental disorder was around two times higher compared to Swedish-born individuals with no mental disorder [40]. The aim of this study was to go one step further and deepen the analyses about pathways to labour market marginalisation specifically among individuals with a common mental disorder. According to the assimilation model, newly arrived migrants can experience labour market marginalisation because of poor language proficiency and scarce knowledge of the culture of the host country [41]. After several years, the level of employment among migrants tends to converge with the host population [41]. In our study, a duration of residence > 10 years among non-refugee migrants and refugees from Africa and Asia positively influenced the risk of unemployment. The duration of residence has been shown to improve the healthcare utilisation rate and intake of psychoactive drugs, which in turn have a positive effect on the health status and the ability to find a job [28]. Also, age at arrival was a significant factor for long-term unemployment in most refugee groups. This is in line with previous studies, which reported that non-European migrants, the most disadvantaged in entering the active labour market, needed more time to reach the level of employment of Swedish-born individuals [11, 41].

### Long-term sickness absence and disability pension

We found a lower risk of long-term sickness absence among refugees and non-refugee migrants from Africa and Asia but not from Europe outside EU25 and South America compared to Swedish-born individuals. Reasons for those discrepancies are to a high extent to be found within the social insurance regulations in Sweden as sickness absence benefits presupposes previous income from work [6]. The risk was particularly low among refugees from Afghanistan and Iraq, countries with a high level of long-term unemployment. Differences in long-term sickness absence with regard to country of birth may also be due to differences in healthcare utilisation. To receive sickness absence benefits, a certificate from a physician is required. This may differ largely between subgroups of migrants [30, 33].

A duration of residence ≥ 10 years was on average associated with a two times higher risk of long-term sickness absence among refugees and non-refugee migrants from Africa and Asia compared to their counterparts with duration of stay  < 10 years. In these populations, age at arrival ≤ 16 years of age was associated with a higher risk of long-term sickness absence compared to those who arrived at > 16 years of age. Thus, it seems that with longer duration of residence in the host country individuals from Africa and Asia become more confident with the Swedish system and/or assimilate with the culture and are thus being able to access the sickness absence benefits. Moreover, with time, these individuals are more likely to enter the labour market, which in turn is a prerequisite for receiving sickness absence [11, 13].

Regarding disability pension, we found a slightly lower risk among non-refugee migrants from Asia and Africa and in some refugee groups compared to Swedish-born individuals. This is in contrast to a previous study in Nordic countries based on the entire population [42]. These discrepancies in findings are most likely due to the differences in study populations, i.e. general population versus young individuals with common mental disorders.

### Strengths and limitations

As strengths of the study, it can be mentioned that we utilised a population-based cohort, using high-quality register data with practically no loss to follow-up. Additionally, we could present data on refugees and non-refugee migrants according to their country of birth and with a long follow-up period which are advantages compared to previous studies. As a limitation, it should be noted that common mental disorders only were defined according to information from in- and specialised outpatient care, but not from primary healthcare. To avoid a potential bias towards more severe morbidity in specialised healthcare, especially considering differences regarding migrants’ healthcare utilisation, we also included participants who had prescriptions for antidepressant medication. Another limitation is that refugees to a lesser extent seek healthcare due to mental disorders compared to persons from the host country [24]. Therefore, we might have captured the migrants with higher severity of the underlying CMD. Finally, to define refugees, we utilised three different reasons of settlement: refugee, humanitarian grounds and need of protection. We did, however, perform sensitivity analyses, which showed similar results for all these groups.

## Conclusions

Young adult refugees and non-refugee migrants with common mental disorders have, compared to Swedish-born individuals with common mental disorders, a higher risk of long-term unemployment. Differences were found between refugees and non-refugee migrants as well as between migrants from different countries. Duration of residence and age at arrival influenced the risk of long-term unemployment, particularly among refugees. The risk of long-term sickness absence was, however, lower in refugees and non-refugee migrants from Africa and Asia compared to Swedish-born individuals, to a large part depending on that eligibility for sickness absence benefit presupposes income from work. Identifying groups of migrant populations at high risk for labour market marginalisation can therefore provide useful information for the design of tailor-made intervention strategies to enhance labour market participation.

## Supplementary Information

Below is the link to the electronic supplementary material.

Supplementary file1 (DOCX 20 KB)

## Data Availability

These data cannot be made publicly available due to privacy regulations. According to the General Data Protection Regulation, the Swedish law SFS 2018:218, the Swedish Data Protection Act, the Swedish Ethical Review Act, and the Public Access to Information and Secrecy Act, these type of sensitive data can only be made available for specific purposes, including research, that meets the criteria for access to this type of sensitive and confidential data as determined by a legal review. Readers may contact Professor Kristina Alexanderson (kristina.alexanderson@ki.se) regarding the data.
