# Supplementary material for: Risk of labour market marginalisation among young refugees and non-refugee migrants with common mental disorders
Source: Soc Psychiatry Psychiatr Epidemiol. 2021 Jan 20;56(6):1025–34. doi: 10.1007/s00127-020-02022-4 (PMC8192389; doi:10.1007/s00127-020-02022-4)
Supplement: Supplementary file 1 — Supplementary file1 (DOCX 20 KB) [file 127_2020_2022_MOESM1_ESM.docx]

**Supplementary table 1.** Countries included in all categorizations.

|  | **Countries included** |
| --- | --- |
| **Asia without Afghanistan, Iran, Iraq and Syria** |  |
|  | Armenia |
|  | Azerbajdjan |
|  | Bahrain |
|  | Bangladesh |
|  | Bhutan |
|  | Brunei |
|  | Cambodia |
|  | China |
|  | Cyprus |
|  | Democratic People’s Republic Korea (North Korea) |
|  | Gaza |
|  | Georgia |
|  | Hongkong |
|  | India |
|  | Indonesia |
|  | Israel |
|  | Japan |
|  | Jordan |
|  | Kazakhstan |
|  | Kuwait |
|  | Kyrgyztan |
|  | Lao |
|  | Lebanon |
|  | Malawi |
|  | Malaysia |
|  | Maldives |
|  | Mongolia |
|  | Myanmar, Burma |
|  | Nepal |
|  | Oman |
|  | Pakistan |
|  | Palestinian territories |
|  | Philippines |
|  | Qatar |
|  | Republic of Korea (South Korea) |
|  | Saudi Arabia |
|  | Sikkim |
|  | Singapore |
|  | Sri Lanka |
|  | South Yemen |
|  | Tajikistan |
|  | Taiwan |
|  | Thailand |
|  | Timor-Leste |
|  | Turkey |
|  | Turkmenistan |
|  | Uzbekistan |
|  | United Arab Emirates |
|  | Vietnam |
|  | West Bank |
|  | Yemen |
| **Africa without Somalia** |  |
|  | Algeria |
|  | Angola |
|  | Benin |
|  | Botswana |
|  | Burkina Faso |
|  | Burundi |
|  | Cameroon |
|  | Cape Verde |
|  | Central African Republic |
|  | Chad |
|  | Comoros |
|  | Congo |
|  | Djibouti |
|  | Democratic Republic of Congo |
|  | Egypt |
|  | Equatorial Guinea |
|  | Eritrea |
|  | Ethiopia |
|  | French Morocco |
|  | Gabon |
|  | Ghana |
|  | Guinea |
|  | Guinea-Bissau |
|  | Ivory Coast |
|  | Kenya |
|  | Lesotho |
|  | Liberia |
|  | Libya |
|  | Madagascar |
|  | Malawi |
|  | Mali |
|  | Mauretania |
|  | Mauritius |
|  | Morocco |
|  | Mozambique |
|  | Namibia |
|  | Niger |
|  | Nigeria |
|  | Rwanda |
|  | Sao Tome & Principe |
|  | Senegal |
|  | Seychelles |
|  | Sierra Leone |
|  | Sudan |
|  | Swaziland |
|  | South Africa |
|  | Tanzania |
|  | The Arab republic of Egypt |
|  | The Gambia |
|  | Togo |
|  | Tunisia |
|  | Uganda |
|  | Zambia |
|  | Zanzibar |
|  | Zimbabwe |
| **South America without Chile** |  |
|  | Argentina |
|  | Bolivia |
|  | Brazil |
|  | Colombia |
|  | Ecuador |
|  | Guyana |
|  | Paraguay |
|  | Peru |
|  | Suriname |
|  | Uruguay |
|  | Venezuela |
| **Europe outside EU25 (without Former Yugoslavia)** |  |
|  | Albania |
|  | Andorra |
|  | Belarus |
|  | Bulgaria |
|  | Gibraltar |
|  | Liechtenstein |
|  | Moldova |
|  | Monaco |
|  | Romania |
|  | Russia |
|  | San Marino |
|  | Switzerland |
|  | Ukraine |
|  | Vatican City |
| **Other countries** |  |
|  | Antigua and Barbuda |
|  | Australia |
|  | Bahamas |
|  | Barbados |
|  | Belize |
|  | Bermuda |
|  | British Virgin Islands |
|  | Canada |
|  | Costa Rica |
|  | Cuba |
|  | Dominica |
|  | Dominican Republic |
|  | El Salvador |
|  | Fiji |
|  | Grenada |
|  | Guatemala |
|  | Haiti |
|  | Honduras |
|  | Jamaica |
|  | Mexico |
|  | Micronesia |
|  | Nauru |
|  | New Guinea |
|  | New Zealand |
|  | Nicaragua |
|  | Panama |
|  | S:t Kitts and Nevis |
|  | S:t Lucia |
|  | S:t Vincent and the Grenadines |
|  | Samoa |
|  | Solomon Islands |
|  | Tonga |
|  | Trinidad and Tobago |
|  | USA |
